# BEHAVIORAL AND PSYCHOLOGICAL SYMPTOMS OF DEMENTIA: DOES GENDER MATTER?

**DOI:** 10.1093/geroni/igz038.2063

**Published:** 2019-11-08

**Authors:** Elizabeth Galik, Marie Boltz, Barbara Resnick, Ann Kolanowski, Kimberly Van Haitsma

**Affiliations:** 1 University of Maryland School of Nursing, Baltimore, Maryland, United States; 2 Pennsylvania State University, State College, Pennsylvania, United States

## Abstract

Regardless of presenting symptoms, there are concerns that BPSD is more often identified in males versus females and males are more likely to be treated with pharmacologic and non-pharmacologic interventions than females. In part this is due to the behaviors in men, specifically aggression, being more distressing for staff and more difficult to manage. The purpose of this study was to test for gender differences in identification and management of BPSD. This was a secondary data analysis using data from the EIT-4-BPSD study including 357 residents, 114 males and 243 females. Men had more aggressive behavior (p=.03) and women more refusal of care (p=.05) and repetitive verbal behavior (p=.03). Men received more mood stabilizers (p=.02) than women. Ongoing research is needed to evaluate if aggression in females may not be recognized or treated as aggressive women are less distressing for staff than these same behaviors in males.

